# Transcriptome analyses of taste organoids reveal multiple pathways involved in taste cell generation

**DOI:** 10.1038/s41598-017-04099-5

**Published:** 2017-06-21

**Authors:** Wenwen Ren, Eitaro Aihara, Weiwei Lei, Nishi Gheewala, Hironobu Uchiyama, Robert F. Margolskee, Ken Iwatsuki, Peihua Jiang

**Affiliations:** 1 Monell Chemical Senses Center, 3500 Market Street, Philadelphia, PA 19104 United States; 2 Department of Molecular and Cellular Physiology, University of Cincinnati, Cincinnati Ohio, OH 45267 United States; 3 Genome Research Center, Tokyo University of Agriculture, Tokyo, 156-8502 Japan; 4 Faculty of Applied Bioscience, Tokyo University of Agriculture, Tokyo, 156-8502 Japan

## Abstract

Taste cells undergo constant turnover throughout life; however, the molecular mechanisms governing taste cell generation are not well understood. Using RNA-Seq, we systematically surveyed the transcriptome landscape of taste organoids at different stages of growth. Our data show the staged expression of a variety of genes and identify multiple signaling pathways underlying taste cell differentiation and taste stem/progenitor cell proliferation. For example, transcripts of taste receptors appear only or predominantly in late-stage organoids. Prior to that, transcription factors and other signaling elements are upregulated. RNA-Seq identified a number of well-characterized signaling pathways in taste organoid cultures, such as those involving Wnt, bone morphogenetic proteins (BMPs), Notch, and Hedgehog (Hh). By pharmacological manipulation, we demonstrate that Wnt, BMPs, Notch, and Hh signaling pathways are necessary for taste cell proliferation, differentiation and cell fate determination. The temporal expression profiles displayed by taste organoids may also lead to the identification of currently unknown transducer elements underlying sour, salt, and other taste qualities, given the staged expression of taste receptor genes and taste transduction elements in cultured organoids.

## Introduction

The sense of taste, initiated by the detection of nutrients or potentially toxic substances by specific receptors expressed in taste cells, plays a critical role in evaluating food before ingesting it^1^. A single taste bud contains about 50~100 elongated taste cells^2^. Based on morphological and functional classification, at least four different types of taste cells are present within single taste buds: type I cells are supporting cells, marked by NTPDaseII; type II cells are receptor cells mediating sweet, bitter, umami, and perhaps other unconventional taste responses (e.g., polycose); type III cells are presynaptic cells, mediating sour taste responses; and type IV cells are precursor cells that express Sonic hedgehog (Shh)^3–5^. In rodent, the average life span of taste cells is estimated to be about two weeks, although this varies somewhat by cell type^6–8^.

Taste cells turn over throughout life and are replenished constantly by adult taste stem/progenitor cells found in the basal area of taste buds or under the trench of the circumvallate papilla^6^. Several recent reports indicate that cells expressing Lgr5 (and/or Lgr6) act as stem/progenitor cells for posterior tongue^9–11^. These cells can give rise to mature taste cells in the oral cavity. Remarkably, in an *in vitro* culture system, single Lgr5^+^ (or Lgr6^+^) cells can generate all three types of mature functional taste receptor cells^11, 12^. Despite a great deal of progress in identifying and characterizing different types of taste cells along with their stem/progenitor cells, the mechanisms underlying this developmental process are largely unknown. *In vivo* studies using knockout or transgenic mouse models indicate a few pathways that are potentially involved in this process. For instance, overexpression of an active form of β-catenin biases multipotent lingual epithelial progenitor cells to differentiate and acquire specific taste cell fates, suggesting that Wnt/β-catenin signaling is involved in taste cell fate determination^13, 14^. Hedgehog (Hh) signaling is also implicated in maintaining taste tissue homeostasis^4, 15, 16^. For example, ectopic expression of Shh can drive *de novo* formation of taste bud cells, while deletion of Gli transcription factors (Hh signaling elements) leads to degeneration of taste buds, and pharmaceutical blockade of Hh signaling leads to altered taste sensation^15, 17–19^.

To systematically survey the genes and pathways involved in generating mature taste cells from stem/progenitor cells, we used an *in vitro* 3-D culture system to grow taste stem/progenitor cells into taste organoids, in which all four types of taste cells are found^11, 12^. We reasoned that, like the native taste system, the differentiation of stem/progenitor cells into mature taste cells in this culture system is regulated by a multitude of genes and pathways in a time-dependent fashion. Here, we describe the temporal profiling of transcriptomes of taste organoids during different stages of growth and identify specific genes and pathways involved in taste cell generation. We found that signaling via Notch, Wnt, Hh, and bone morphogenetic proteins (BMPs) can modulate the growth and differentiation of taste organoids.

## Results

### Tracking the generation of taste cells using marked taste organoids

**Figure 1:**
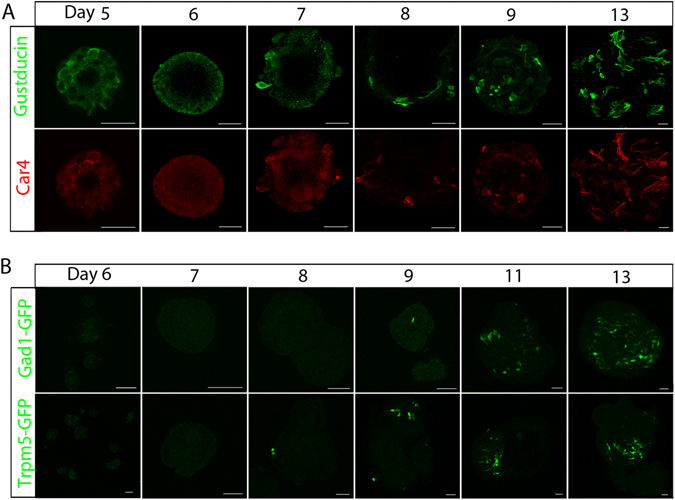
Tracking the generation of taste cells in cultured organoids. (**A**) Whole-mount immunostaining of cultured taste organoids (days 5–13) derived from sorted Lgr5^+^ cells. Gustducin-immunoreactive cells were detected in organoids by day 7 (top row). Car4-immunoreactive cells were detected in organoids by day 8 (bottom row). (**B**) Intrinsic GFP^+^ cells in organoids derived from Gad1-GFP mice (top row) and Trpm5-GFP mice (bottom row) 6–13 days after dissociation and seeding. GFP^+^ cells were readily visible by day 8 or 9. Scale bars, 100 µm. Experiments were performed in triplicate.

We used immunostaining to determine when taste stem/progenitor cells in cultured taste organoids begin to differentiate into taste cells that express taste receptors or taste transduction elements in cultured taste organoids. Because of technical challenges in performing immunostaining of early-stage organoids, we performed whole-mount staining of organoids grown from sorted Lgr5^+^ or Lgr6^+^ taste stem/progenitor cells from day 5 on (Fig. 1). Immunostaining for the type II cell marker gustducin^20^ and the type III cell marker carbonic anhydrase 4 (Car4)^21^ showed that immunoreactive cells can be detected as early as day 7 or 8 (Fig. 1A). To follow the generation of taste cells in real time, we generated organoids from transgenic mice that express green fluorescent protein (GFP) under the control of the promoters of Trpm5 (type II cell marker)^22, 23^ or Gad1 (type III cell marker)^24, 25^. We previously succeeded to generate taste organoids directly from isolated circumvallate papilla tissue^12^, and others have reported that dissociated lingual epithelial cells can generate organoids^26^. However, most of these organoids appear to be non-taste organoids, as taste cell markers could not be detected^26^.

We dissociated circumvallate papilla epithelium into single cells from Trpm5-GFP or Gad1-GFP adult mice (8~12 weeks), plated them in our Matrigel-based 3-D culture system, and followed the GFP signal. In all cases, we found that only a small portion of freshly dissociated cells were GFP positive (Fig. S1A). As expected, the GFP signal disappeared a day or so after plating, indicating that dissociated mature taste cells died quickly in cultures. Starting around day 8 or 9, the GFP signal became visible in many organoids (up to 80% of organoids at day 14; Fig. S1A, B), indicating that organoid cells started to differentiate into mature taste cells. Thus, during the growth of taste organoids, the differentiation and maturation of type II (Trpm5-GFP) and type III (Gad1-GFP) cells appear to take a week or so and stabilize around days 12–14 (Fig. 1B). This developmental time line largely mirrors that of these types of cells in native tissue revealed by lineage tracing analyses^8, 9^.

### Taste organoid transcriptomes at different stages of growth

To understand the transcriptional regulation of the ontogeny of mature taste cells and to determine the genes and pathways involved in the generation of taste bud cells, we profiled the transcriptional landscapes of taste organoids at different stages. Single Lgr5-GFP^+^ cells were sorted and divided into 14 different wells (Fig. S2). Starting day 2 (freshly dissociated cells designated as day 0), organoids were collected each day until day 14, and RNA was extracted. We generated cDNAs from organoids at days 2, 4, 6, 8, 10, 12, and 14 and performed RNA-Seq (RNA sequencing). Two independent experiments were performed to generate RNA-seq data. To identify temporal expression patterns, we ran hierarchical clustering and K-means clustering algorithms to cluster the RNA-Seq data (datasets 1 and 2) in reads per kilobase of transcript per million mapped reads (RPKM). RPKM data were then trimmed (Supplementary Files 1 and 2) by setting inclusion criteria as follows: (1) an RPKM read is ≥0.2 for day 14 organoids, to remove low or no expressed genes; and (2) this set of genes also has a sum of reads of organoids at all different stages >0.2 for dataset 2 (see below) for cross-comparison. This led to 14,124 genes for analysis.

**Figure 2:**
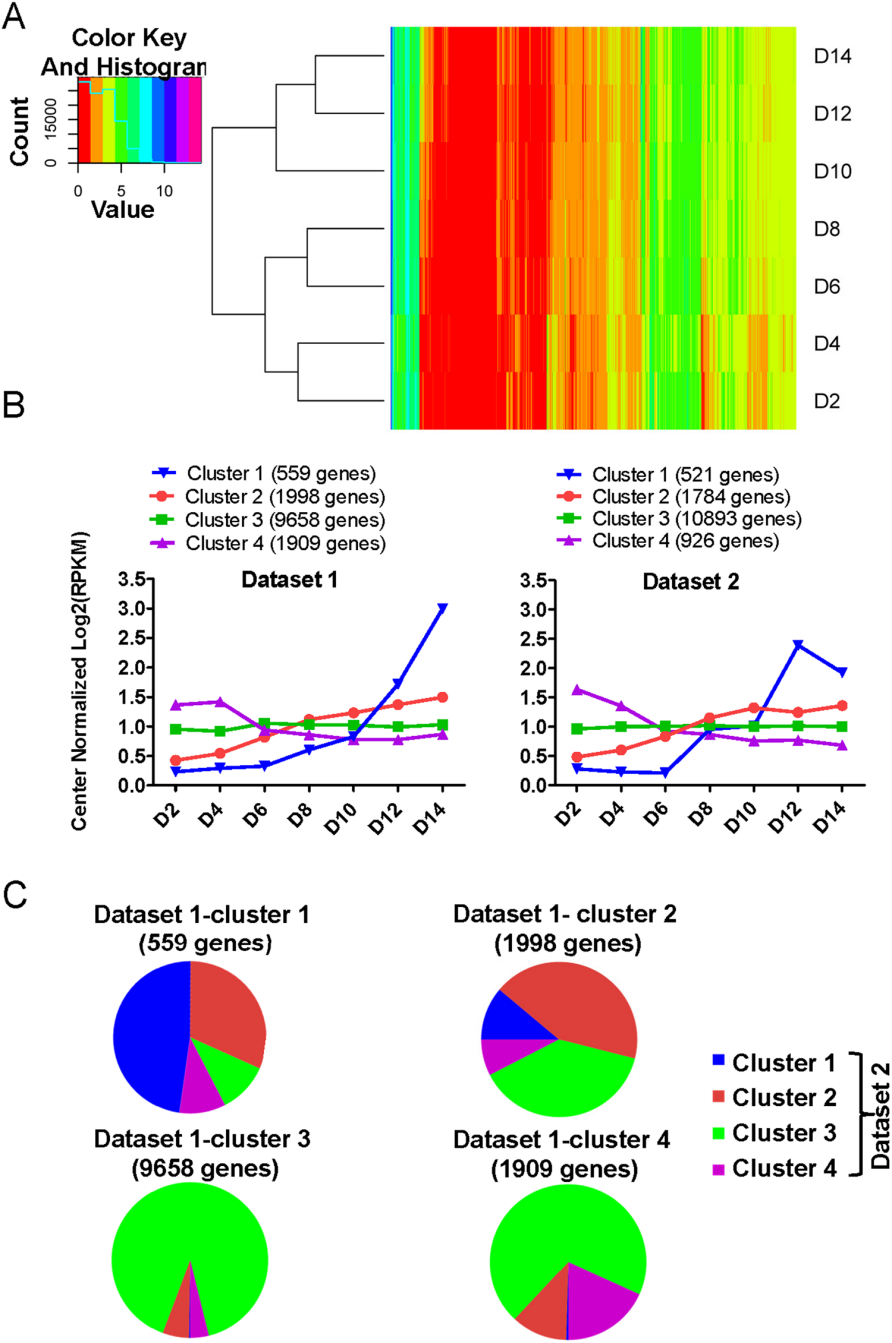
Temporal expression patterns of genes during organoid development. Transcriptome analyses were performed on day (D) 2, 4, 6, 8, 10, 12, and 14 organoids derived from sorted Lgr5^+^ cells. (**A**) Hierarchical cluster analysis shows that organoids at closely related stages are clustered together based on their gene expression patterns. The heat map indicates distinct gene expression profiles for organoids at each developmental stage. The color key represents log_2_-transformed RPKM counts. (**B**) K-means analysis reveals four different gene clusters in datasets 1 and 2 with their unique temporal expression patterns. Taste-related genes such as Tas1rs and Tas2rs are predominantly found in cluster 1, showing an increased expression level during organoid growth, especially at later stages. The y-axis depicts normalized log_2_(RPKM) values for centers for each cluster. (**C**) Relative portion of interacting genes between gene clusters derived from K-means analyses of datasets 1 and 2.

As expected, hierarchical clustering of datasets 1 and 2 revealed that organoids at closely related growth stages clustered together due to similarity in their gene expression profiles (Figs 2A and S3). The dendrograms of both datasets also showed two root branches, one formed by organoids at days 2, 4, 6, and 8 and the other by organoids at days 10, 12, and 14, indicating general differences between early-stage and late-stage organoids. That is concordant with the appearance with taste cells expressing taste cell markers (e.g., Trpm5-GFP^+^ cells, Gad1-GFP^+^ cells) around day 8 in cultured organoids (Fig. 1).

To determine which genes may be involved in taste cell generation (e.g., differentiation) and function (e.g., receptors, channels), we performed K-means analysis to cluster genes with similarity in their temporal expression to find patterns in our datasets. We mined the dataset 1 by setting K at different values and found that the four-clusters (*K* = 4) model provides biologically interpreted information and agrees with a simple model of taste cell generation. The taste cell generation process starts with taste stem/progenitor cells, these cells subsequently give rise to precursor cells (e.g., Shh^+^ cells), and then precursor cells differentiate into mature taste cells (e.g., Tas1r3^+^ cells). Given this model, we expected that a subset of genes relating to taste cell fate determination (e.g., *Shh*) would be upregulated during the precursor stages, and another subset of genes (e.g., *Tas1r3*) would be upregulated during the maturation stages. A majority of genes would show no change in their expression during the growth of organoids because they are not directly related to taste cell fate determination or taste cell function, such as house-keeping genes; and another set of genes would be potentially downregulated (e.g., cell cycle genes), presumably due to a reduction in the percentage of proliferating cells in the late-stage organoids, with an concomitant increase in the number of the newly generated and differentiated cells. All 14,124 genes from Dataset 1 that met our criteria for inclusion were clustered into four different clusters with distinct temporal expression patterns (Fig. 2B, left; see also Supplementary File 3). The expression levels of genes in cluster 1 (559 genes from dataset 1) were low or not detectable at very early stages but increased rapidly during the growth of late-stage organoids. Mature taste cell markers (e.g., *Tas1r1*, *Tas1r2*, *Tas1r3*, *Tas2r102*, *Tas2r104*, *Tas2r105*, *Tas2r108*, *Tas2r113*, *Tas2r115*, *Tas2r116*, *Tas2r118*, *Tas2r126*, *Tas2r135*, *Tas2r137*, *Tas2r138*, *Gng13*, *Trpm5*, *Pkd1l3*, *Car4*, *Trcg1*, *Gad1*) were well represented in this cluster. The enrichment of taste-related genes in this cluster was further confirmed by functional annotation clustering using DAVID bioinformatics resources (Supplementary File 4). The term “taste transduction” is ranked first with a p-value of 2.9E-15 and Benjamini of 5.8E-13 by the KEGG pathway analysis implemented in DAVID^27, 28^. All other terms (80 total; e.g., renin secretion, calcium signaling pathway, amoebiasis, GABAergic synapse) had a p-value ≥ 1.2E-2 (Supplementary File 4).

Genes in cluster 2 (1,998 genes from dataset 1) also showed an increase in the expression level during organoids growth, albeit with a faster rate of increase in early-stage organoids than in late-stage organoids. Notably, additional taste transduction components and other signaling pathway components known to regulate taste cell specification and function are present in this cluster, such as Gustducin (*Gnat3*), Sonic hedgehog (*Shh*), Keratin 20 (*krt20*), *Kcnq1*, *Pkd2l1*, Enac channel (*Scnn1a*, *Scnn1b*, *Scnn1g*), *Bdnf*, *Gnb3*, *Gna14*, *Ptch1*, *Wnt11*, *Kcnj2*, Gpr120 (*Ffar4*), *Plcβ2*, *Ncam1*, *Pou2f3*, *Hcn1*, *Notch3*, *Dll3*, *Dll4*, and Ntpdase-2 (*Entpd2*). By the KEGG analysis, terms (228) such as gastric acid secretion (p-value: 5.0E-6, Benjamini: 1.3E-3), cholinergic synapse (p-value: 1.1E-5, Benjamini: 1.4E-3), morphine addiction (p-value: 5.9E-5, Benjamini: 5.2E-3), and pathways in cancer (p-value: 1.4E-4, Benjamini: 8.9E-3) were associated with this cluster (Supplementary File 4).

Cluster 3 includes genes showing little or no change during the growth of organoids, suggesting that this set of genes may not be directly involved in differentiation or function pertinent to taste cells. A majority of genes (9,658 of 14,124 genes analyzed from dataset 1) were found in this cluster. Another set of 1,909 genes comprised cluster 4, showing a substantial decrease in their expression level between days 4 and 6 and then maintaining their expression level afterward. The KEGG analysis found 177 terms. The top five terms from this list of chart records were Fanconi anemia pathway (p-value: 3.8E-16; Benjamini: 8.2E-14), DNA replication (p-value: 1.7E-14; Benjamini: 2.1E-12), pyrimidine metabolism, mismatch repair, and cell cycle (Supplementary File 4). The apparent downregulated expression of this set of genes (mostly associated with proliferation) could be simply because late-stage organoids comprise differentiating and differentiated cells, compared with proliferating cells predominantly present in early-stage organoids.

To further confirm growth-stage-dependent expression of genes and pathways found in each cluster in dataset 1, we performed RNA-Seq analysis of another set of samples extracted from organoids grown from a different batch of sorted Lgr5^+^ cells (dataset 2). Similar to dataset 1, hierarchical clustering and K-mean clustering were performed on trimmed dataset 2 (Supplementary Files 2 and S3). Overall, the results were similar between datasets 1 and 2. K-means clustering yielded four clusters (Supplementary File 5) with patterns and number of genes similar to those of dataset 1. Because of inherent noise of each dataset, as well as the data-mining nature of K-means analysis, we applied stringent criteria to remove potentially falsely classified genes and to identify only genes present in corresponding clusters in both datasets. The pie charts in Fig. 2C show the intersecting genes between clusters of dataset 1 and 2. Cluster 1 of dataset 1 (559) and cluster 1 of dataset 2 (521) share 268 genes (including most of taste receptors); 856 genes were common to cluster 2 of dataset 1 (1,998) and of dataset 2 (1,784); cluster 3 of dataset 1 (9,658) and of dataset 2 (10,893) share 8,732 genes; cluster 4 of dataset 1 (1,909) and of dataset 2 (926) share 348 genes.

When we combined clusters 1 and 2 (both sets of genes show increased expression during organoid growth), 1,523 genes were common to both datasets (dataset 1: 559 cluster 1 + 1,998 cluster 2, 60%; dataset 2: 521 cluster 1 + 1,784 cluster 2, 66%) (Supplementary File 6). The KEGG pathway analysis again showed that “taste transduction” (p-value, 8.1E-13; Benjamini: 2.0E-10) tops the list of 205 chart records.

To further determine if the genes clustered by the K-means analysis show statistically significant changes in expression during organoid growth, we performed likelihood ratio tests (ANOVA-like tests) to estimate differential gene expression across all stages of organoid growth from two datasets, based on a negative binominal generalized linear model (GLM)^29^. Out of the 14124 genes, 2616 genes show differential expression during organoid growth (Supplementary File 7). Out of the 1523 genes that are grouped in Cluster 1 and 2 in both datasets, 871 (57%) show significant change (P ≤ 0.05, after FDR correction) in expression during organoid growth (Supplementary File 7). The KEGG pathway analysis again showed that “taste transduction” (p-value, 2.5E-12; Benjamini: 5.9E-10) tops the list of 186 chart records (Supplementary File 8), which also include gastric acid secretion, insulin secretion, cholinergic synapse, Rap1 signaling pathway, Ras signaling pathway, chemokine signaling pathway, Notch signaling pathway, axon guidance, and Wnt signaling pathway, among many others. Many of these genes may play important yet undetermined roles in taste/perigemmal cell differentiation and taste cell function (e.g., G protein-coupled receptors: *Gpr37*, *Gpr162*, *Gpr137c*, *Gpr6*, *Olfr461*, *Adgrb1*, *Adgrb2*, *Oprd1*; ion channels: *Tmc5*; transmembrane proteins: *Tmem171*, *Tmem212*, *Tmem163*, *Tmem45a*, *Tmem38a*, *Tmem211*, *Tmem150c*, *Tmem191c*, *Tmem221*, *Tmem132e*; transcription factors: *Nkx2-2*, *Ascl1*, *Ascl2*, *Ovol3*; axon guidance: *Sema5a*, *Epha4*, *Epha5*). Ovol3 has not been characterized in any system previously. To confirm its expression in taste tissues and to further validate our RNA-seq analyses, we performed reverse-transcription PCR and showed *Ovol3* is strongly expressed in the circumvallate papillae but not in the surrounding non-taste lingual epithelial tissue (Fig. S4). Out of the 348 genes that are grouped in Cluster 4 in both datasets, 215 genes show significant changes in expression (P ≤ 0.05, after FDR correction) (Supplementary File 7). Again, DNA repair and cell-cycle-related genes (e.g., *Fanca*, *Brca1*, *Eme1*, *Cdc6*, *Cdc7*, *Chek1*) are enriched (e.g., KEGG terms: Fanconi anemia pathway, homologous recombination, cell cycle, Base excision repair) (Supplementary File 8).

Altogether, our datasets provide a rich and nearly comprehensive list of genes related to taste cell differentiation as well as taste function and warrant further study.

### The roles of the Wnt, BMP, Hh, and Notch signaling in the growth and differentiation of taste organoids

Our RNA-Seq data showed the Wnt, BMP, Hh, and Notch signaling elements was either continuously expressed at different stages or specifically upregulated at certain stages. For instance, Dll3, Dll4, Notch4, Bmp2, Fzd4, Shh, Ptch1, and Ptch2 were found in either cluster 1 or cluster 2 of both datasets. Essentially following the general temporal expression pattern of taste signaling elements, their expression levels increased steadily in a time-dependent manner. On the other hand, other signaling components of these pathways show some variability between datasets 1 and 2 in terms of temporal expression. Nevertheless, transcripts for many of these signaling components were readily detectable throughout organoid growth. The Wnt, BMP, Shh, and Notch signaling pathways are known to be involved in tissue development and stem cell maintenance for a wide variety of tissues^15, 30–32^. To assess how these pathways are involved in the growth and differentiation of taste organoids, we used pharmacological approaches to determine their functions in taste cell generation and organoid growth.

### Wnt signaling

**Figure 3:**
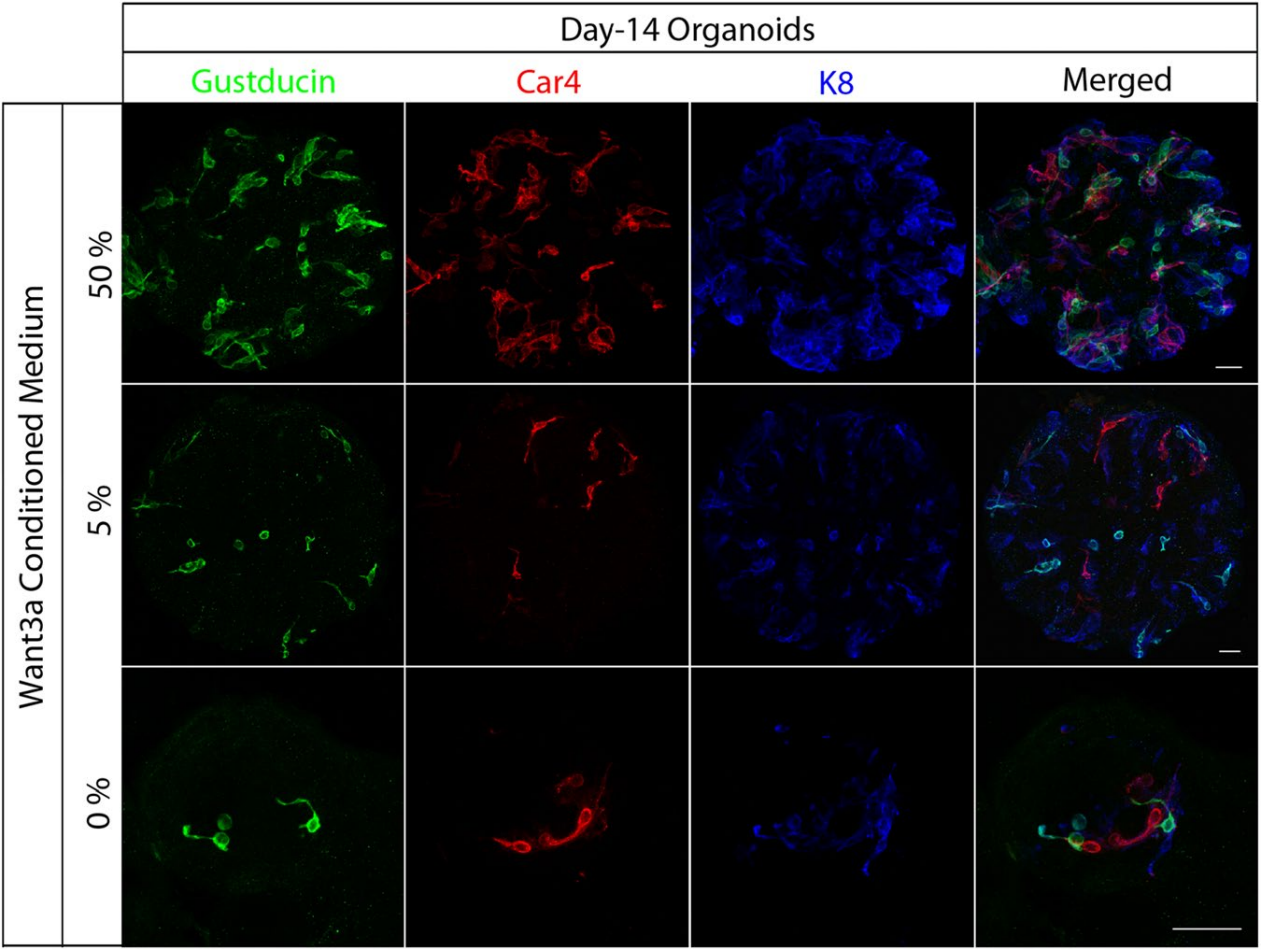
Wnt-dependent generation of taste cells in cultured organoids. Day-14 organoids derived from sorted Lgr5-GFP^+^ cells were immunostained in whole mount with anti-Gustducin (green), anti-Car4 (red), and anti-K8 (blue) antibodies. Organoids were cultured in 50% (top row), 5% (middle row), or 0% (bottom row) Wnt3a conditioned medium. Scale bars, 100 µm. Representative images shown; at least three independent experiments were performed.

To determine the role of Wnt signaling in our culture system, organoids were generated from sorted Lgr5-GFP^+^ cells and cultured in Wnt3a conditioned medium (CM) with high (50%), low (5%), or no (0%) Wnt3a. Immunostaining of day-14 organoids with antibodies against Gustducin, Car4, NTPDaseII and K8 (Keratin-8) showed Wnt-activity-dependent generation of taste cells (Figs 3 and S8). More taste cells (Type I, Type II, and Type III) were stained in organoids cultured in medium with high (50%) than low (5%) or no (0%) Wnt3a (Figs 3 and S8). To more readily see the effect of the Wnt3a CM, we dissociated circumvallate papilla epithelium from Gad1-GFP mice and cultured them to generate organoids in the presence (50%) or absence (0%) of Wnt3a CM. Organoids cultured with Wnt3a CM grew much larger than those without Wnt3a CM (Fig. S5A, B). Moreover, the percentage of organoids with intrinsic GFP signals was much higher in the presence of Wnt3a CM, that was, 76.34% ± 2.93 of organoids were GFP^+^ (Gad1-GFP), compared to 11.89% ± 1.29 GFP^+^ organoids without Wnt3a CM (Fig. S5C). The Wnt activity of the Wnt3a CM was assessed by the TOP-flash assay (Fig. S5D). Together, these results indicate that stimulation of Wnt signaling enhances taste cell differentiation in cultured taste organoids.

### BMP signaling

**Figure 4:**
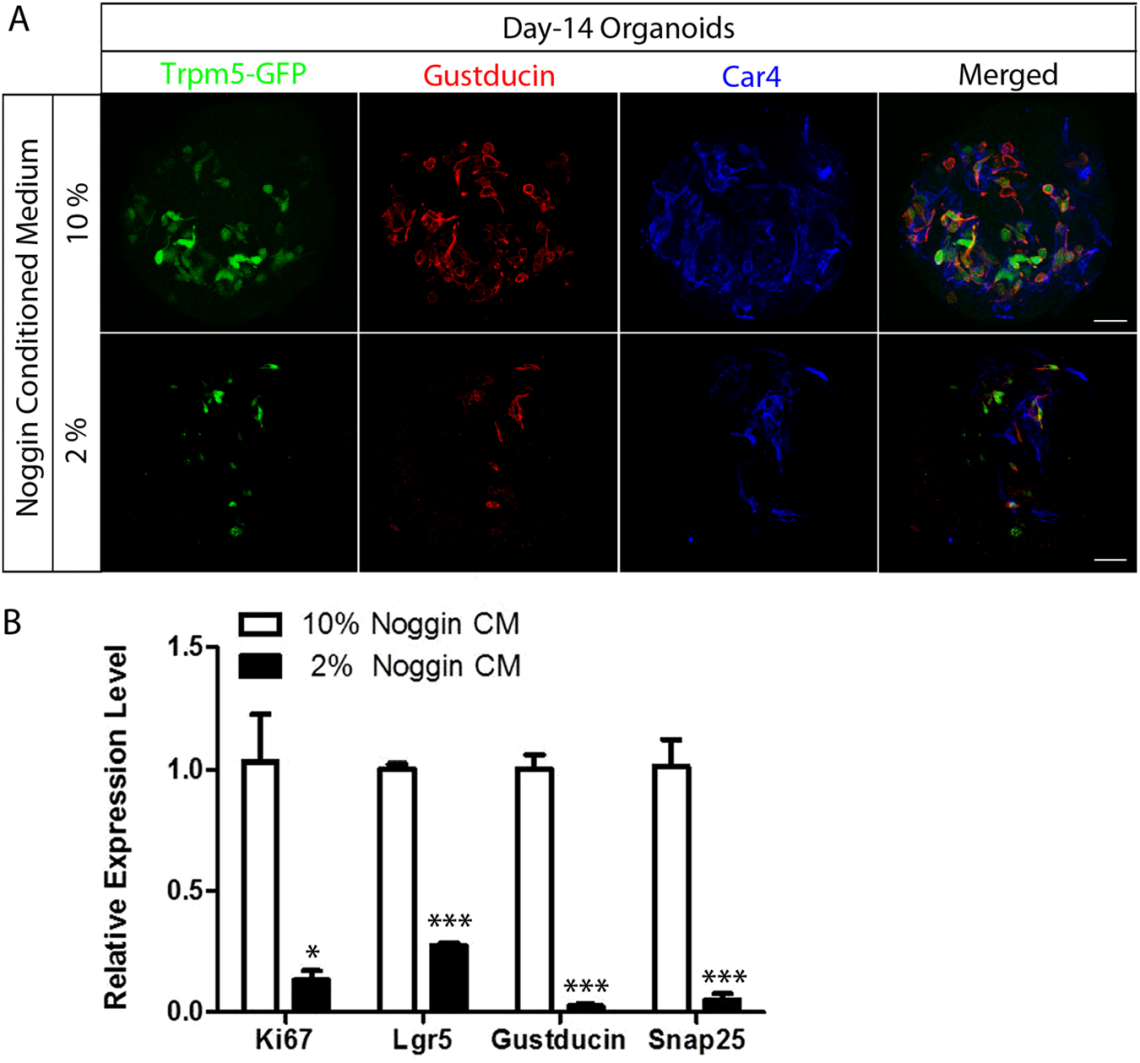
Noggin promotes the proliferation and differentiation of taste organoids. (**A**) Day-14 organoids derived from Trpm5-GFP mice were immunostained in whole mount with anti-Gustducin (red) or anti-Car4 (blue) antibodies or visualized by intrinsic Trpm5-GFP fluorescence (green). Organoids were cultured in medium supplemented with 10% (top row) or 2% (bottom row) Noggin conditioned medium (CM). Scale bars, 100 μm. (**B**) Quantitative real-time PCR analysis of the relative expression level of genes specific for proliferation (Ki67), stem cells (Lgr5), and taste cells (Gustducin and Snap 25) in organoids cultured for 14 days in 10% or 2% Noggin CM. Experiments were performed in triplicate. *p < 0.05, ***p < 0.001.

To determine the role of BMP signaling in our taste organoid culture system, we used Noggin, an extracellular antagonist that can inhibit BMP action^31, 33^. Removal of Noggin from culture medium leads to loss of Lgr5 expression and proliferation arrest in cultured intestinal organoids^34^. We generated organoids from single dissociated cells from Trpm5-GFP mice and then cultured them in medium supplemented with either 10% or 2% Noggin CM. More taste cells (e.g., Trpm5-GFP^+^, Gustducin-immunoreactive^+^, Car4-immunoreactive^+^ cells) were detected in organoids grown in 10% Noggin CM than in 2% Noggin CM (Fig. 4A), and the percentage of organoids with intrinsic Trpm5-GFP cells reached 90% in 10% Noggin CM (Fig. S6A). This increase in the numbers of type II and type III cells in 10% Noggin CM was consistent with our quantitative real-time PCR results (Fig. 4B), where the expression levels of Gustducin and Snap25 (type III cell marker) were significantly reduced in lower percent of Noggin CM (2%). A similar pattern was observed for Lgr5 and the proliferating cell marker Ki67 (Fig. 4B). Organoids generated from sorted Lgr5^+^ cells cultured in medium supplemented with different amounts of Noggin CM (20%, 10%, 0%) also showed similar results. Organoids cultured in 20% Noggin CM grew larger than those cultured in 10% Noggin CM (Fig. S6B, C). Our immunostaining results showed that a great number of cells in organoids grown under both conditions were immunoreactive for taste cell markers. As expected, when Noggin CM was absent from the culture medium, there were significantly decreased numbers of mature taste cells as well, including NTPDaseII^+^ type I cells (Figs S6D and S8). These data suggest that Noggin plays an essential role in taste cell differentiation and stem cell proliferation in the cultured taste organoids.

### Notch signaling

**Figure 5:**
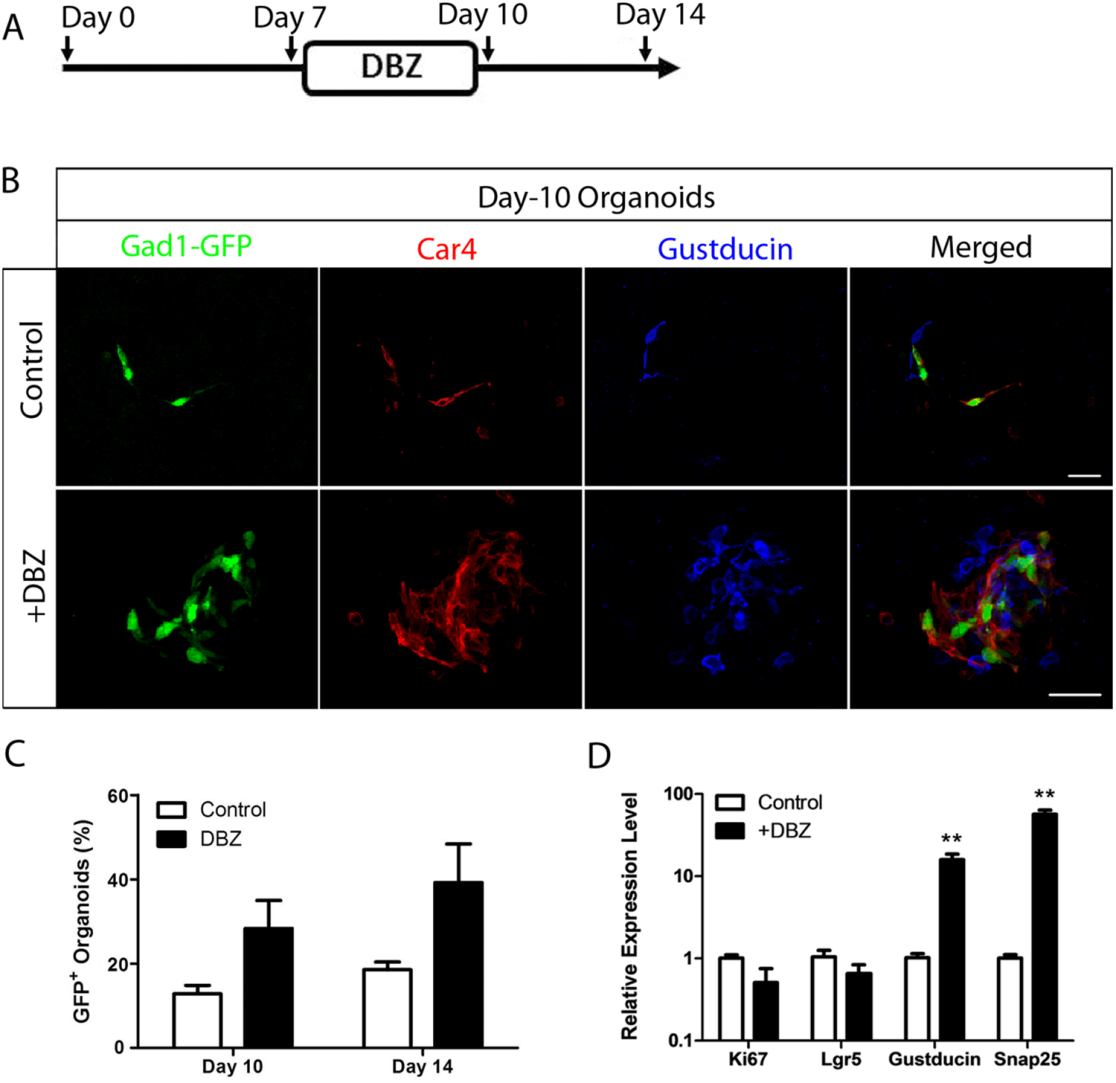
Dibenzazepine (DBZ) accelerates and promotes differentiation of taste cells in cultured organoids. (**A**) Schematic illustration of DBZ treatment on cultured organoids at indicated days. (**B**) Day-10 organoids derived from Gad1-GFP mice were immunostained in whole mount with anti-Car4 (red) or anti-Gustducin (blue) antibodies or visualized by intrinsic Gad1-GFP fluorescence (green) without (control, top row) or with (bottom row) DBZ treatment. Scale bars, 100 µm. (**C**) Percentage of organoids with Gad1-GFP^+^ cells with (10 days, n = 45; 14 days, n = 53) or without (10 days, n = 36, p = 0.09036; 14 days, n = 48, p = 0.08975) DBZ treatment. (**D**) Quantitative real-time PCR analysis of the relative expression level of genes specific for proliferation, stem cells, and taste cells in control and DBZ-treated organoids. **p < 0.01. Experiments were performed in triplicate.

To determine the role of Notch signaling in the generation of taste cells, we used the specific γ-secretase inhibitor dibenzazepine (DBZ) to block cleavage of the Notch receptor^35, 36^, just before the appearance of taste cells (about day 7) (Fig. 5A). Using organoids generated from Gad1-GFP mice, we found that adding DBZ (10 µM) between days 7 and 10 accelerated the differentiation of Gad1-GFP^+^ cells in taste organoids (Fig. 5B). At day 10, there was a significant increase in the number of Gad1-GFP^+^ cells as well as Gustducin^+^ and Car4^+^ cells, revealed by immunostaining (Fig. 5B). The number of organoids containing Gad1-GFP cells increased at both day 10 and day 14, although this was not statistically significant (Fig. 5C). By quantitative real-time PCR analysis, we found that expression levels of Gustducin and Snap were highly increased in DBZ-treated organoids, while Ki67 was relatively unchanged (Fig. 5D). Similar results were obtained with organoids generated from Trpm5-GFP mice or Lgr5^+^ cells, either at day 10 or day 14 (data not shown). To determine if the increase in the number of Gustducin^+^ and Car4^+^ cells were due to conversion of other types of taste cells to type II and type III cells, immunostaining was performed on organoids at day 14 with antibodies against type I taste cell marker NTPDaseII, the number of organoids that contain NTPDaseII^+^ cells was significantly reduced after DBZ treatment (Fig. S8). These data suggest that blocking Notch signaling accelerates the differentiation of mature taste cells and alters cell fate determination as well.

### Hh signaling

**Figure 6:**
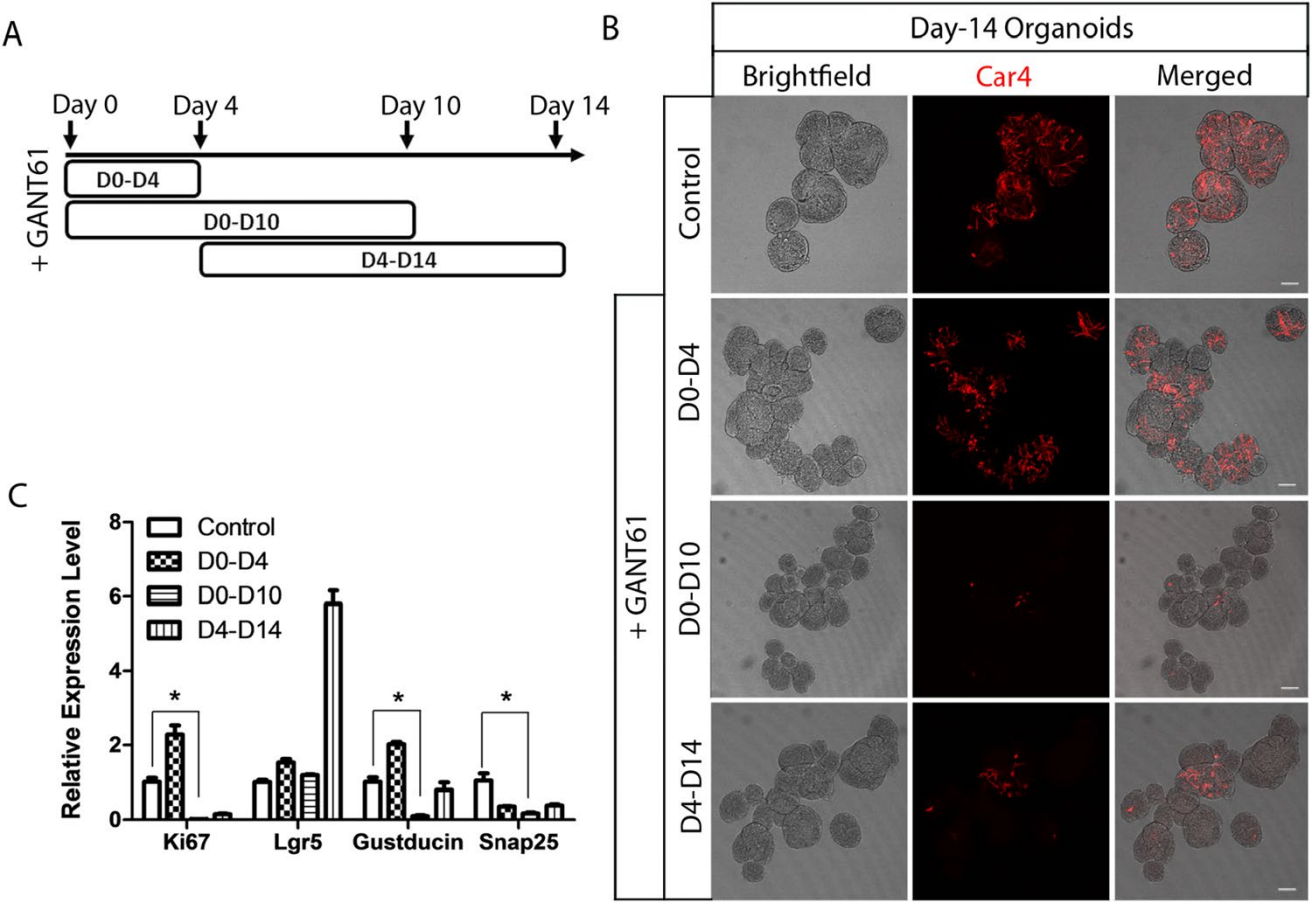
GANT61 arrests the growth of taste organoids. (**A**) Schematic illustration of GANT61 treatment on cultured organoids at different stages. (**B**) Immunostaining of day-14 organoids with anti-Car4 antibody without (top row) or with (lower rows) GANT61 treatment at different stages. Scale bars, 100 µm. (**C**) Quantitative real-time PCR analysis of the relative expression level of genes specific for proliferation, stem cells, and taste cells in control and GANT61-treated organoids. *p < 0.05. Experiments were performed in triplicate.

To determine if Hh signaling is required for the growth and differentiation of taste organoids, we used a specific Gli transcriptional factor inhibitor, GANT61, to block Hh signaling during different stages of organoid growth^37^. We cultured organoids derived from Gad1-GFP mice and added GANT61 (20 µM) into the medium at different stages of culturing (Fig. 6A). When GANT61 was added into freshly isolated cells for 4 days, organoids did not form (Fig. S7A), suggesting that GANT61 either arrested the proliferation of stem cells or induced cell death. To distinguish between these two possibilities, we removed GANT61 at day 5 to determine if cells would proliferate and generate organoids after the removal of GANT61. We observed that single cells started to grow into organoids after removal of GANT61, and these organoids included mature taste cells at day 14 (Fig. 6B) but were smaller than the untreated organoids (Fig. S7B). Furthermore, long-term incubation with GANT61 from day 0 to day 10 inhibited the growth of organoids, which had much smaller size and fewer taste cells (Fig. 6B; see also Fig. S7A).

Quantitative analysis performed at day 14 showed that both proliferating cell marker Ki67 and mature taste cell markers Gustducin and Snap25 were significantly downregulated by GANT61 from day 0 to day 10 (Fig. 6C). However, 10 days of incubation with GANT61 in a later stage from day 4 to day 14 had much less of an effect (Fig. 6C; see also Fig. S7A). These organoids had fewer taste cells and smaller size than controls but more taste cells and larger size than those incubated with GANT61 from day 0 to day 10, suggesting that blocking Shh pathway by GANT61 arrested the proliferation of stem cells in cultured taste organoids, especially in the early stage of culturing. Interestingly, the level of Lgr5 expression significantly increased in organoids treated with GANT61 from day 4 to day 14, suggesting an interplay between Wnt signaling and Hh signaling.

## Discussion

We analyzed the transcriptomes of taste organoids at different stages of development and found that in organoids at earlier stages multiple cell-cycle-related genes are activated, including those for M phase, cell cycle, mitosis, and cell division, suggesting that active proliferation occurs in the earlier organoids. At later stages (e.g., days 12–14), organoids express a multitude of taste-specific genes, including T1R and T2R receptors, indicating time-dependent maturation of taste cells. Multiple genes and pathways appear to be involved in the growth and differentiation of taste stem/progenitor cells in cultured organoids. Using specific pharmacological agents, we demonstrated critical roles of Hh, Wnt, BMP, and Notch signaling in proliferation and active cycling of taste stem/progenitor cells. Moreover, we found that modulation of Notch signaling accelerates the maturation of taste receptor cells in taste organoids.

### Signaling Pathways Detected

We combined RNA-Seq with a novel culture system to uncover genes and pathways involved in the ontogeny and cell-fate determination of taste cells. Different waves of transcriptional activity were evident at different stages of development of taste organoids, suggesting specific and fine regulation of genetic networks in the patterning and generation of taste receptor cells. For instance, transcripts of transcription factors that are known to specify taste cell determination (e.g., *Pou2f3*, *Ascl1*, *Ascl2 Nkx2-2*) were generally upregulated slightly prior to those of taste receptors (e.g., Tas1rs, Tas2rs), agreeing with their roles in taste cell fate specification. Detailed analyses of these upregulated genes (e.g., 871 out of 1,523 genes common to both datasets in clusters 1 and 2) and regulatory networks *in vivo* and *in vitro* may provide a deeper understanding of the regenerative process and tissue homeostasis of taste bud cells throughout life, along with taste transduction and connectivity with innervating nerves.

We showed that several signaling pathways are involved in the growth and differentiation of taste organoids. Hh signaling is implicated in taste tissue homeostasis^4, 15, 38^. Not surprisingly, we found that the blockade of Gli transcription led to the arrest of growth of taste organoids. The involvement of Shh signaling may occur mainly in proliferation of taste stem/progenitor cells. In developing taste tissue, Shh negatively regulates Wnt activity^39^. It is noteworthy that upregulation of Lgr5 transcripts occurred by blockage of Shh signaling during day 4–14 of culture (Fig. 6). This phenomenon fits with the proposal that Shh suppresses the Wnt/β-catenin pathway during taste papillae development^39^.

Wnt signaling, especially the canonical Wnt/β-catenin signaling pathway, is essential for maintaining self-renewal of stem cells^40, 41^. Inhibition of Wnt signaling ablated long-term organoid cultures^42^. Here, we found that organoid growth and taste cell generation were Wnt activity dependent.

BMPs are secreted members of the transforming growth factor β family of signaling molecules, which regulate a wide range of developmental processes, including stem cell and organ formation^31^. Our RNA-Seq data showed that the pattern of BMP activation/inactivation changes drastically between days 2 and 6 of organoid culture. This change in pathway activation was similar to what we observed in Wnt/β-catenin signaling. Noggin activity can affect the growth of organoids as well as taste cell generation.

Because of their increased expression as organoids mature, a subset of genes related to Notch signaling (e.g., Dll3, Dll4, Notch4) had similar temporal expression patterns to taste-related molecules (Supplementary Files 3 and 5). Notch signaling is implicated in multiple developmental processes^32, 41, 43^ and in taste tissue differentiation and homeostasis^32, 44^. However, the role of Notch signaling in mammalian taste development and regeneration is largely unexplored. We showed that DBZ can accelerate the maturation of type II and type III taste receptor cells in cultured taste organoids, and concomitantly reduce the number of NTPDaseII^+^ type I cells. This indicates that Notch signaling may be involved in taste cell fate determination. Most likely, the increase in the number of type II and type III taste cells is due to fate alteration of type I cells in cultured taste organoids in the presence of DBZ. Future work using knockout models and organoid cultures may provide a mechanistic understanding of the Notch signaling pathway in taste tissue homeostasis.

Multiple chemotherapy agents are known to affect taste function, presumably by affecting taste cell regeneration. Given the effects of GANT61 on taste cell proliferation and recycling, this culture system may be used as a prescreening tool to evaluate the potential side effects of cancer therapy drugs.

### Temporal expression of taste genes

Previous attempts to identify receptors, channels, and key signaling elements of taste transduction used cDNA libraries to identify genes enriched in taste tissue^45, 46^. Although these methods helped identify the receptors for sweet and umami^47^, the detectors responsible for transducing high salt, sour, and some other unconventional tastes remain elusive. RNA-Seq analysis of taste organoids provides a temporal dimension to the identification of candidate genes relevant for taste transduction. In our dataset, most known taste receptors and signaling elements appear only in late-stage organoids (cluster 1). Thus, the transducers for high-salt, sour, and other tastes may also be expressed only in late-stage organoids. Analysis of the function of late-expressed channels or receptors may help reveal the identity of additional taste receptors.

The growth and differentiation of cultured organoids appear to largely mimic the *in vivo* regeneration of native taste bud cells. Nevertheless, our cultured organoids differ from native tissue. For instance, we observed the expression of only a subset of Tas2r bitter receptor genes in our dataset and failed to detect many other Tas2rs, possibly because (a) their expression level is below our detection limit due to the depth of sequencing—indeed, it appears that those receptors are only weakly expressed in native taste tissue^48^; (b) organoids require more time for maturation in our system; (c) our system cannot entirely capture everything happening in native taste buds; or (d) technical difficulties in amplifying all relevant genes from our organoids due to a limited amount of starting material and potential biases during the amplification of cDNA for sequencing. Despite a great number of mature taste cells in our cultured organoids, we did not observe well-organized taste bud-like structures *in vitro*, suggesting some limitation of our cultured system. Further optimization is required for growing a fully functional bud *in vitro*.

### Experimental Procedures

#### Transgenic mice

Genetically engineered mice (*Lgr5-EGFP-ires-CreERT2*; stock no. 008875; Gad1-GFP, stock no. 007677) were obtained from the Jackson Laboratory. Trpm5-GFP mice were generated in the Margolskee lab. All experiments were performed under National Institutes of Health guidelines for the care and use of animals in research and approved by the Institutional Animal Care and Use Committee of the Monell Chemical Senses Center.

#### Cell sorting

To obtain Lgr5^+^ cells, tongues from *Lgr5-EGFP-ires-CreERT2* ^+/−^ mice were injected with ~0.7 mL of an enzyme mixture containing dispase (2 mg/mL, Roche) and collagenase (1 mg/mL, Roche) in Tyrode’s solution (145 mM NaCl, 5 mM KCl, 10 mM HEPES, 5 mM NaHCO_3_, 10 mM pyruvate, 10 mM glucose) for 15 min at 37 °C. Tongue epithelium was peeled gently off from the underlying connective tissue, and the regions surrounding the circumvallate and foliate papillae were dissected out and collected. Tissues were then minced using scissors and digested by trypsin (0.25%) for 30 min at 37 °C. DMEM/10% FBS (1 mL) medium was added to stop trypsinization. Digested tissues were spun down by centrifugation at 1,200 rpm for 10 min. With HBSS buffer (5 mM MgCl_2_, 10 mM HEPES, 50 µg/mL DNase, 0.1 g/mL bovine serum albumin, 10 µg/mL 4′,6-diamidino-2-phenylindole (DAPI), 5% fetal bovine serum (FBS)) in the tube, tissues were mechanically dissociated into single cells using a fire-polished glass pipette. Single-cell suspensions were filtered using 70-µm nylon mesh (BD Falcon no. 352350) to remove large aggregates, followed by further filtering with 35-μm nylon mesh (BD Falcon no. 352235). Cells were purified using fluorescence-activated cell sorting (Flow Cytometry and Cell Sorting Resource Laboratory, University of Pennsylvania), according to the green fluorescent protein signal (excitation, 488 nm; emission, 530 nm). Red fluorescent protein channel (582 nm) was used to gate out the autofluorescent cells, and DAPI (450 nm) was used to gate out dead cells (Fig. S2).

#### Cell isolation without sorting

To generate taste organoids from Trpm5-GFP and Gad1-GFP mice, the same dissociation procedure was used. Cells were dissociated from the circumvallate papilla tissue from at least three mice. After being filtered twice using 70- and 30-μm nylon mesh sequentially, cells were plated directly onto low-attachment 24-well plates at a density of about 1 × 10^5^ cells/well (Corning Ultra-Low Attachment Plates, Fisher Scientific), with 5% chilled Matrigel (Corning no. 356231) in 0.4 mL culture medium for each well.

#### Noggin conditioned medium

A full-length cDNA clone that was engineered to express Noggin with a Myc-DDK tag was obtained from OriGene (no. MR225276) and subcloned into pCDNA3.1-Zeocin. A stable line was established after selection of HEK-293 (pEAK Rapid) cells that were transfected with pCDNA3.1-Noggin-Myc-DDK using Zeocin. The expression of Noggin was confirmed by immunostaining. Cells were grown into confluence and then cultured in OptiMEM for additional 7 days prior to harvest as Noggin conditioned medium (CM).

#### 3-D organoid culture

The complete culture medium was based on 20% DMEM/F12 medium (Life Technologies no. 11320033), 50% Wnt3a CM (generated from a Wnt3a-producing cell line, gift from Dr. Hans Clevers, selected by 125 µg/mL Zeocin), 20% R-spondin CM (generated from an R-spondin cell line, a gift of Dr. Jeffery Wittsett, selected by 600 µg/mL Zeocin), and 10% Noggin CM (generated from pEAK Rapid cell line, selected by 400 µg/mL Zeocin), supplemented with epidermal growth factor (50 ng/mL; Peprotech no. 315-09), N2 (1%; Life Technologies no. 17502-048), B27 (2% (vol/vol); Life Technologies no. 17504044), and penicillin-streptomycin (1×; ThermoFisher Scientific no. 15140122). For the freshly dissociated single cells, Y-27632 (10 µM; Sigma no. Y0503) was added in the medium to prevent dissociation-induced apoptosis, and 5% chilled Matrigel was used for organoid growth. For organoids used for RNA-Seq and the Notch pathway study, commercial Noggin (10 ng/mL; Peprotech no. 250-38) was used prior to our establishment of the Noggin-producing stable line. The medium was first changed after 5 days of culturing and then every 2–3 days based on the density of growing organoids.

#### Determination of Wnt activity

Wnt activity was determined by TOP-flash assay, as described previously^49^. Briefly, HEK-293T cells were transfected using linear polyethylenimine (MW 25,000; Polysciences no. 23966), with luciferase reporter of β-catenin-mediated transcriptional activation (TOP-flash vector) or its control luciferase reporter, FOPflash (mutant of TOP-flash). (These reporter constructs were kindly shared by Dr. Clevers). A pRL *Renilla* luciferase control reporter vector (Promega) served as a control for transfection efficiency. Then, transfected HEK-293T cells were treated with different concentrations of Wnt3a and/or R-spondin CM for 24 h. Cells were lysed, and cell lysates were assayed for firefly luciferase and *Renilla* luciferase activity on a luminometer using the Dual-Luciferase reporter assay system (Promega, ref E1910). TOP-flash and FOP-flash luciferase activities were normalized to *Renilla* luciferase activity.

#### RNA-Seq analysis

Total RNA was extracted from organoids using Nucleospin RNA XS kit (Clontech no. 740902.50). Then, a SMART-Seq v4 Ultra Low Input RNA Kit for Sequencing (Clontech no. 634888) was used to generate and amplify full-length cDNA. cDNAs were sheared by a S2 sonicator (Covaris). Next, sequencing libraries were generated by KAPA Hyper Prep Kits for Illumina (Kapa Biosystems). Libraries were sequenced on an Illumina HiSeq 2500 to generate 100-bp reads. Sequence reads obtained after quality control with filtering were mapped to GRCm38 (mm10), and gene expression levels were calculated with the RNA-Seq mapping algorithm in CLC Genomics Workbench 8.0.3 (CLC Bio). Statistical tests based on a negative binominal generalized linear model (GLM) similar to that of edgeR were conducted in CLC genomics workbench 9.5.3 with Advanced RNA-Seq plugin. We used likelihood ratio tests (ANOVA-like tests) to estimate differential gene expression across days while controlling for difference between replications. The read data have been deposited to the DNA Data Bank of the Japan Sequence Read Archive (accession no. DRA005238).

#### Clustering analysis and GO analysis

For K-means clustering, the trimmed datasets (see main text) were log transformed, and then the reads for each gene across all different stages were normalized to the average of reads of all different stages for each gene. The log-transformed (RPKM + 1), normalized datasets were analyzed using the K-means algorithm implemented in R with four centers. Iteration max was set to 30,000, and nstart was set to 30, followed by KEGG analysis using the DAVID web tool (http://david.abcc.ncifcrf.gov/) for each set of gene clusters. For hierarchical analysis, we used the hclust and heatmap.2 functions in R to analyze the log-transformed (RPKM + 1) and trimmed datasets to determine the hierarchical relationship among organoids at different developmental stages.

#### Whole-mount immunostaining

Immunostaining of organoids was performed in 1.5-mL Eppendorf tubes. After being washed with PBS twice, organoids were fixed with 4% paraformaldehyde in PBS, supplemented with 5 mM MgCl_2_, 10 mM EGTA, and 4% (wt/vol) sucrose for 15 min at room temperature. Fixed organoids were incubated in blocking buffer (SuperBlock (Thermo Scientific PI-37525), 2% (vol/vol) donkey serum, 0.3% Triton X-100) for 1 h after being washed 3 times with PBS. Organoids were then incubated with primary antibodies overnight at 4 °C. After three washes with PBS, organoids were incubated with appropriate secondary antibodies for 2 h, and nuclear counterstaining was performed with DAPI (10 µg/mL, Life Technologies) for 10 min at room temperature. Organoids in mounting medium were then transferred onto slides for imaging (Prolong Gold Antifade Mountant, Thermo Scientific). Primary antibodies were rat anti-K8 (1:10; Developmental Studies Hybridoma Bank; RRID no. AB_531826), rabbit anti-Gustducin (1:500; Santa Cruz; RRID no. AB_673678), goat anti-Car4 (1:20; R&D Systems no. AF2414), and rabbit anti-NTPDaseII (1:500; Centre de Recherche du CHUL, Quebec, Canada). Secondary antibodies were donkey anti-rabbit: Alexa Fluor 488 (Abcam no. ab150073), Alexa Fluor 555 (Abcam no. ab 150074), and Alexa Fluor 647 (Abcam no. ab 150075); donkey anti-goat: Alexa Fluor 555 (Abcam no. ab 150130) and Alexa Fluor 647 (Abcam no. ab150131); and donkey anti-rat: Alexa Fluor 647 (Abcam no. ab 150155). Confocal images were compressed z-stacks (~15 μm) and acquired by a Leica SP2 confocal microscope. Fluorescence images were acquired by a Nikon Eclipse E800 microscope.

#### Quantitative real-time PCR and reverse transcription PCR

Organoids were collected at indicated days, and RNAs were purified with PureLink RNA Mini Kit (Fisher Scientific no. 12183018A). SuperScript VILO Master Mix was used to generate cDNA (Fisher Scientific no. 11755050). Fast SYBR Green Master Mix (Fisher Scientific no. 4385612) was used to run real-time PCR. StepOne Software, version 2.3, was used for data analysis. Primer sets were, for Gustducin : forward, CATGGCTACACTGGGGATTG; reverse, GATTTCAGCCAGCTGTGGAG; Snap25: forward, ACCTAGGAAAATTCTGCGGG; reverse, CTGGCCACTACTCCATCCTG; Ki67: forward, TCTGATGTTAGGTGTTTGAG; reverse, CACTTTTCTGGTAACTTCTTG; Lgr5: forward, TAAAGACGACGGCAACAGTG; reverse, GATTCGGATCAGCCAGCTAC; and β-actin: forward, GATTACTGCTCTGGCTCCTA; reverse, ATCGTACTCCTGCTTGCTGA.

For RT-PCR, the circumvallate papilla and the surrounding non-taste epithelial tissues were harvested from wildtype mice. RNA and cDNA were prepared as described above for RT-PCR. Primer sets were, for Ovol3: forward, ATGCCCAGGGTCTTTCTTGTGAGGA; reverse, TCAGGTCGTGCGGTGTAGGGTGCGG; for GAPDH: forward, GCATGGCCTTCCGTGTTCCTA; reverse. GATGCCTGCTTCACCACCTTCT. The PCR product of Ovol3 was confirmed by sequencing.

#### Pathway studies

To study the Notch signaling pathway, dibenzazepine (DBZ; 10 µM; YO-01027, Selleckchem no. S2711) was added to medium at day 7 of culturing for 4 days. At day 10, the medium with DBZ was removed and organoids were either harvested or cultured with fresh medium until day 14.

To study the Hh signaling pathway, GANT61 (20 µM; Selleckchem no. S8075) was added into medium at indicated days. To remove GANT61, the medium was removed by centrifugation and fresh medium without GANT61 was added.

To study the Wnt and Noggin signaling pathways, Wnt3a or Noggin CM was either removed or reduced from the culture medium by substituting with an equal volume of DMEM/F12 or DMEM/FBS (FBS does not have an apparent effect on organoid growth and differentiation) (Supplementary Figure 9).

### Statistical analysis

Unless specified, two-tailed Student’s t-tests were performed for statistical analysis. Data are presented as mean ± standard error of mean.

## Electronic supplementary material

Supplementary Information

Dataset 1

Dataset 2

Supplementary file 1

Supplementary file 2

Supplementary file 3

Supplementary file 4

Supplementary file 5

Supplementary file 6

Supplementary file 7

Supplementary file 8
