# Supplementary material for: Transcriptome analyses of taste organoids reveal multiple pathways involved in taste cell generation
Source: Sci Rep. 2017 Jun 21;7:4004. doi: 10.1038/s41598-017-04099-5 (PMC5479815; doi:10.1038/s41598-017-04099-5)
Supplement: Supplementary file 1 — Supplementary Information [file 41598_2017_4099_MOESM1_ESM.pdf]

Supplementary Information:

Transcriptome analyses of taste organoids reveal multiple pathways involved in taste cell generation

Wenwen Ren[1], Eitaro Aihara[2], Weiwei Lei[1], Nishi Gheewala[1], Hironobu Uchiyama[3], Robert F. Margolskee[1], Ken Iwatsuki[4]*, Peihua Jiang[1]*

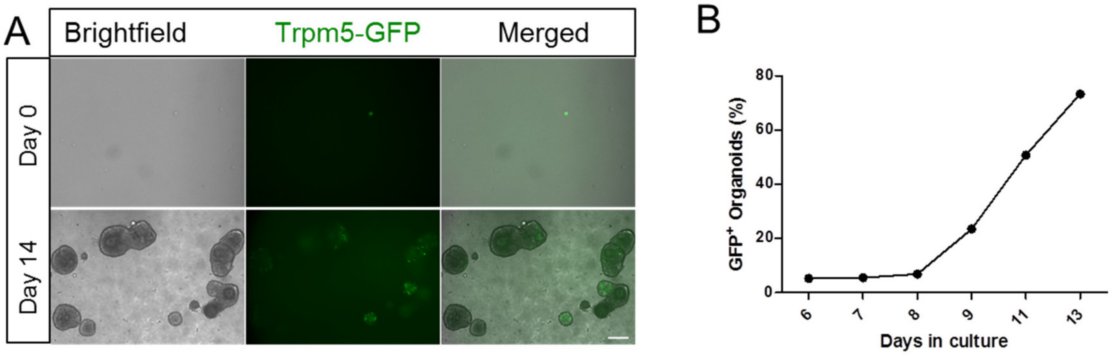

Fig. S1. GFP$^+$ cells in organoids from Trpm5-GFP transgenic mice.

A, Top row: Representative image of single dissociated cells from digested tongue epithelium from Trpm5-GFP mice. Some dissociated cells expressed Trpm5-GFP fluorescence (green). Bottom row: Representative low-magnification image of Trpm5-GFP organoids cultured for 14 days. Scale bars, 100 µm. B, Percentage of organoids showing Trpm5-GFP$^+$ cells during development. Experiments were performed at least three times.

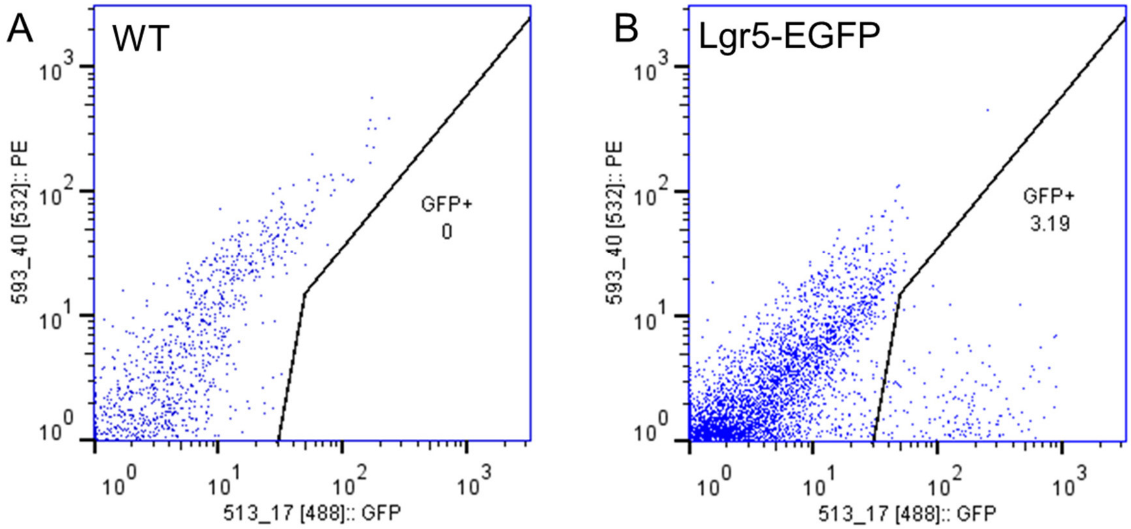

Fig. S2. **Flow sorting of dissociated single Lgr5-EGFP$^+$ cells.**

Representative results of fluorescence-activated cell sorting of cells isolated from taste tissue from wild-type (WT; A) and Lgr5-EGFP$^+$ (B) mice.

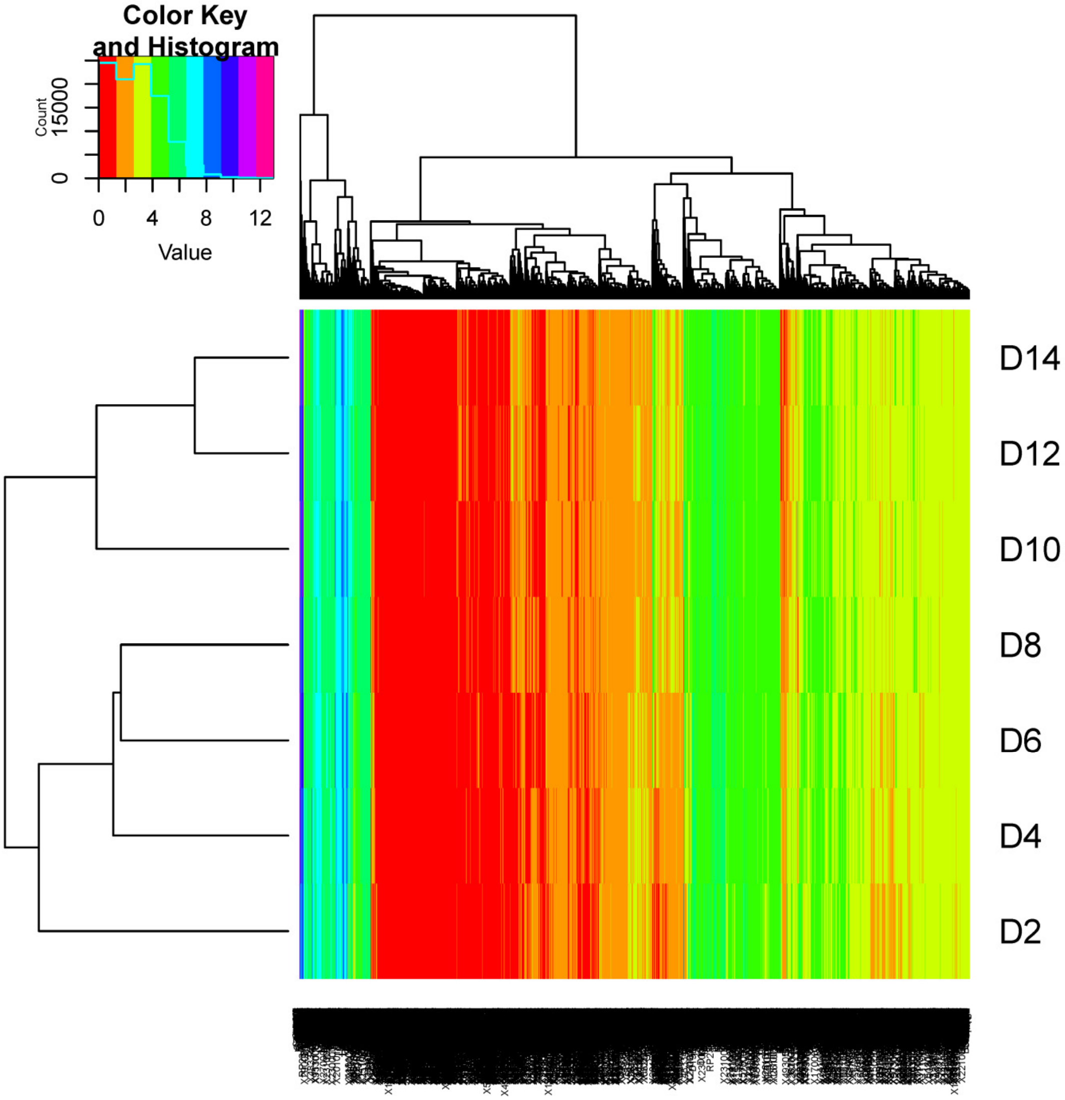

Fig. S3. Hierarchical cluster analysis of dataset 2

Dendrogram shows that organoids at closely related stages are clustered together based on their gene expression profiles. The heat map indicates distinct gene expression profiles for organoids at each developmental stage. The color key represents $\log_2$-transformed RPKM counts.

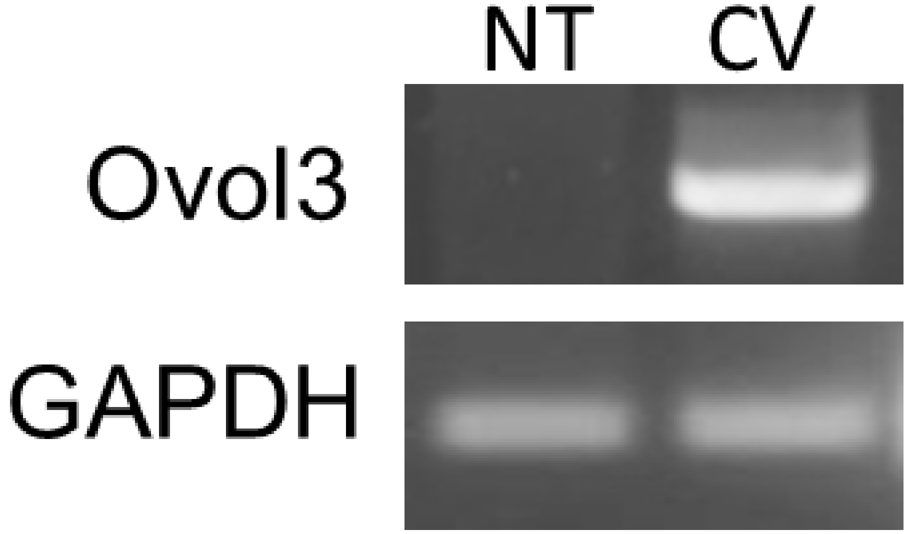

Fig. S4. RT-PCR analysis of *Ovol3*

RT-PCR demonstrates *Ovol3* expression (570 bp) in circumvallate (CV) papilla taste tissue but not in the surrounding non-taste (NT) lingual epithelium. The *GAPDH* RT-PCR controls confirm equivalent template amounts from CV and NT cDNA.

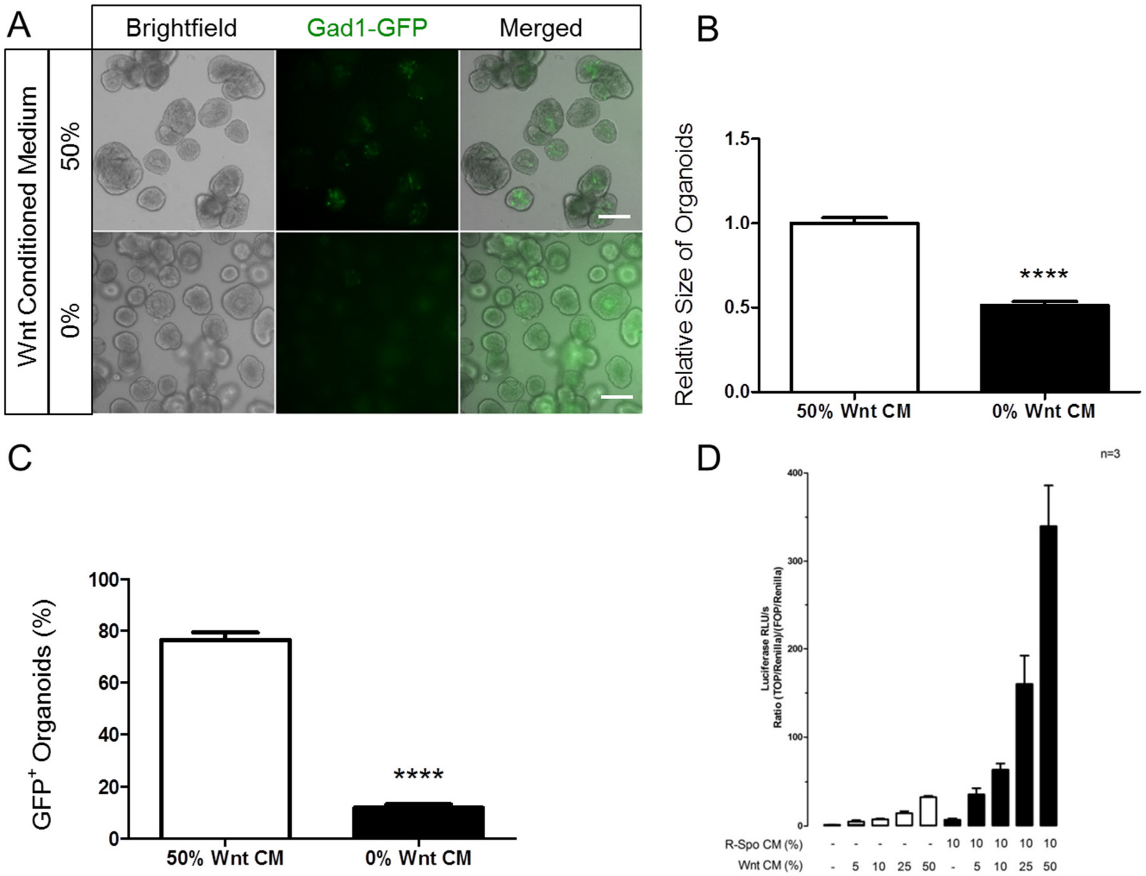

Fig. S5. Removal of Wnt reduced growth and differentiation of taste organoids.

A, Representative low-magnification images of Gad1-GFP organoids cultured for 14 days with (top row) or without (bottom row) Wnt conditioned medium (CM). Scale bar, 100 µm. B, Relative size (mean ± SD) of day-14 organoids with (1.000 ± 0.03262, n=51) or without (0.5115 ± 0.02228, n=54) Wnt CM. C, Percentage (mean ± SD) of day-14 organoids with Gad1-GFP$^+$ cells with (76.34 ± 2.934%; n=76) or without (11.89 ± 1.293%; n=156) Wnt3a CM. D, TOP-flash assay of different percentages of Wnt CM in the absence or presence of R-spondin CM (mean ± SD). **** $p < 0.0001$. Experiments were performed in triplicate or quadruplicate.

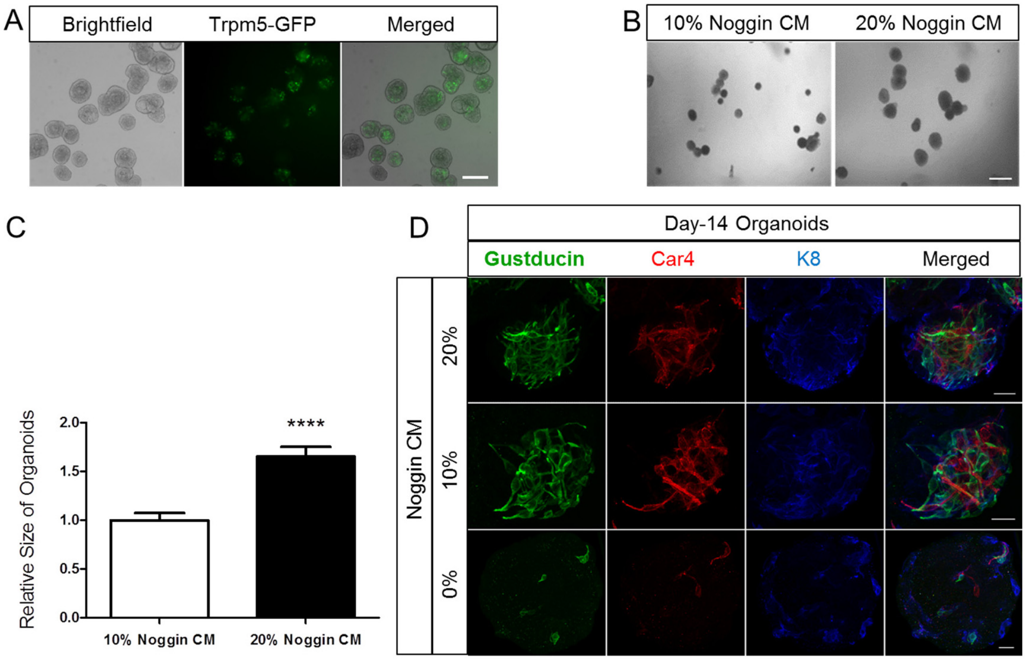

Fig. S6. Noggin promotes the proliferation and differentiation of taste organoids.

A, Representative images of day-14 organoids with Trpm5-GFP cells cultured with 10% Noggin conditioned medium (CM). Scale bar, 100 µm. B, Representative bright-field low-magnification images of day-14 organoids derived from Trpm5-GFP mice cultured in 10% or 20% Noggin CM. Scale bar, 200 µm. C, Relative size (mean ± SD) of day-14 organoids cultured in 10% (1.000 ± 0.07737, n=31) or 20% (1.655 ± 0.09915, n=26) Noggin CM. Data were collected from two independent preparations. **** $p < 0.0001$. D, Whole-mount immunostaining of day-14 organoids derived from sorted Lgr5-GFP^{+} cells with anti-Gustducin (red), anti-Ca4 (blue), and anti-K8 (blue) antibodies cultured in 20% (top row), 10% (middle row), or 0% (bottom row) Noggin CM. Scale bars: top and middle row, 20 µm; bottom row, 100 µm. Experiments were performed in triplicate.

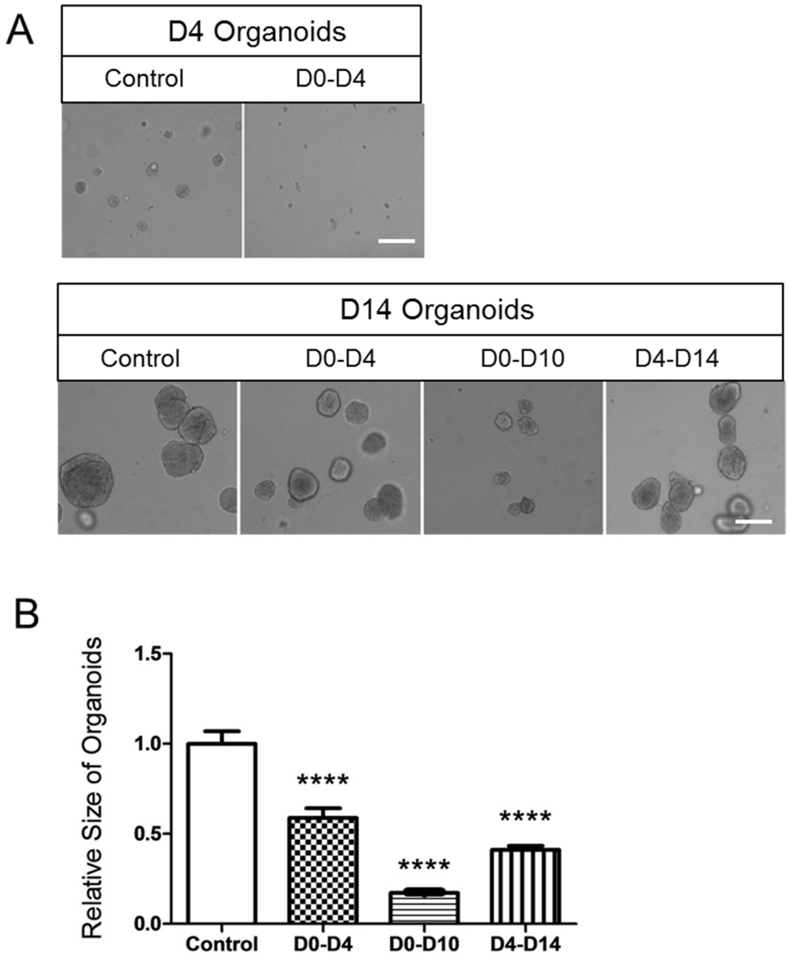

Fig. S7. Addition of GANT61 arrests the growth of taste organoids.

A, Representative bright-field images of organoids cultured with or without GANT61 for different days. Scale bars, 100 µm. B, Average relative size (mean ± SD) of day-14 organoids cultured without (control, 1.000 ± 0.06918, n=81) or with GANT61 treatment at different stages (D0-D4, 0.5887 ± 0.05305, n=71; D0-D10, 0.1717 ± 0.005642, n=81; D4-D14, 0.4105 ± 0.02250, n=87). **** $p < 0.0001$. Data were collected from two independent preparations.

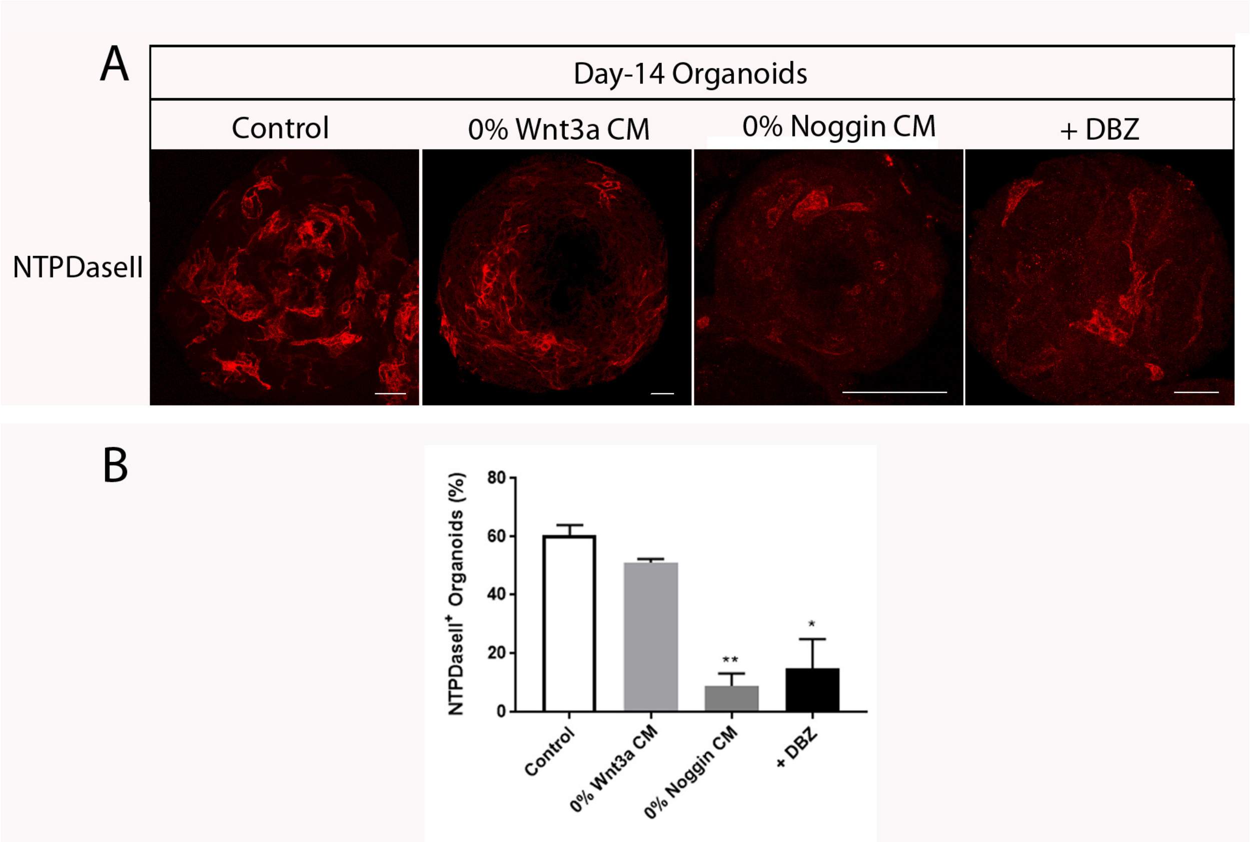

Fig. S8. Wnt3a, Noggin and DBZ affect the differentiation of NTPDaseII^{+} type I cells.

A, Representative images of day-14 organoids cultured under different conditions and immunostained with anti-NTPDaseII antibody. Fewer NTPDaseII^{+} cells were detected in the absence of Wnt3a CM or Noggin CM or in the presence of DBZ. Scale bars: 100 μm. B, The percentage (mean ± SD) of day-14 organoids showing immunoreactivity for NTPDaseII under different culture conditions： control (60.5 ± 2.5, n=72), absence of Wnt3a CM (51% ± 1, n=41), absence of Noggin CM (9% ± 3, n=51) and presence of DBZ (15% ± 7, n=68). * $p < 0.05$, ** $p < 0.01$. Data were collected from two independent preparations.

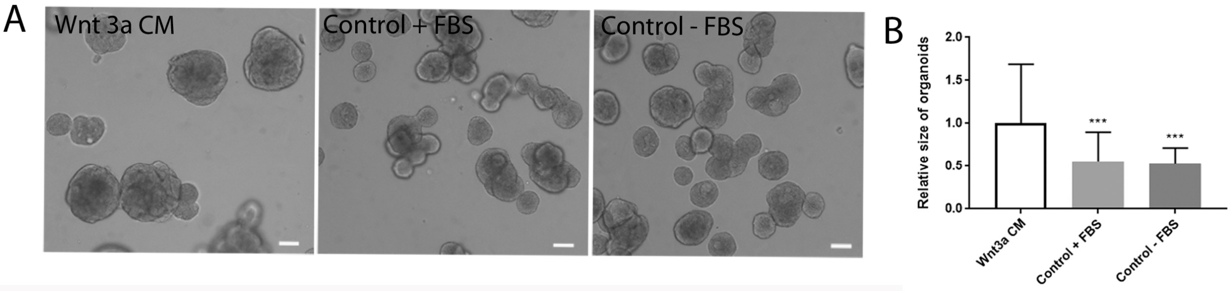

C

	Wnt3a CM	DMEM/FBS	DMEM/F12	R-spondin CM	Noggin CM
Wnt3a CM	50%	0%	20%	20%	10%
Control + FBS	0%	50%	20%	20%	10%
Control - FBS	0%	0%	70%	20%	10%

Figure S9. The effect of Wnt3a CM on organoid growth is due to Wnt3a not FBS.

A) Representative images of organoids grew under different culture conditions. Scale bars: 100 µm. B) The relative size of organoids grew under Wnt3a CM (1 ± 0.1088, n=40) or control conditions with (0.558 ± 0.05462, n=39) or without FBS (0.5312 ± 0.02974, n=37). Without Wnt3a CM, organoids grew much smaller; no differences were detected for organoids grew in the presence of or absence of FBS. *** $p < 0.001$. C) Culture medium compositions. Data were collected from two preparations.

Dataset 1: RNA-Seq data of the first batch of organoids in RPKM.

Dataset 2: RNA-Seq data of the second batch of organoids in RPKM.

Supplementary file 1: Trimmed dataset 1.

Supplementary file 2: Trimmed dataset 2.

Supplementary file 3: List of genes in each of four clusters, generated by K-means analysis of normalized, log-transformed, trimmed dataset 1.

Supplementary file 4: The results of KEGG analysis of genes in each K-means cluster.

Supplementary file 5: List of genes in each of four clusters, generated by K-means clustering of normalized, log-transformed, trimmed dataset 2.

Supplementary file 6: List of 1,523 genes intersecting clusters 1 and 2 of datasets 1 and 2, showing upregulated expression as organoids grow, and list of 348 genes intersecting cluster 4 of datasets 1 and 2, showing downregulated expression in organoids between day 4 and day 6.

Supplementary file 7: The results of ANOVA-like tests of 14124 genes from trimmed dataset 1&2. The results of the 1523 upregulated genes grouped in Clusters 1 and 2 from both datasets shown in worksheet “Cluster1&2 Diff Exp”, and the results of the 348 downregulated genes grouped in Cluster 4 from both datasets shown in worksheet “Cluster4 Diff Exp”.

Supplementary file 8: The results of KEGG analysis of genes filtered after ANOVA-like tests ($P \leq 0.05$) from Cluster1&2 or from Cluster 4.
